# One-Tube RPA-CRISPR/Cas12a Assays for Rapid and Visual Detection of *Pseudomonas fluorescens* and *Bacillus cereus*

**DOI:** 10.3390/foods15061059

**Published:** 2026-03-17

**Authors:** Changli Yang, Gaoke Wang, Xiaowu Zhou, Jie Song, Xu Luo, Hua Liu, Haijuan Zeng, Wenhui Wu, Xiaoyan Zhao, Jinbin Wang

**Affiliations:** 1 School of Food Science and Technology, Shanghai Ocean University, Shanghai 201306, China; 13792536860@163.com (C.Y.);; 2 Key Laboratory of Agricultural Genetics and Breeding, The Biotechnology Research Institute, Shanghai Academy of Agricultural Sciences, Shanghai Professional Technology Service Platform of Agricultural Biosafety Evaluation and Testing, Shanghai 201106, China; 3 Key Laboratory for Safety Assessment (Environment) of Agricultural Genetically Modified Organisms, Ministry of Agriculture and Rural Affairs, Shanghai 201106, China

**Keywords:** *Bacillus cereus*, *Pseudomonas fluorescens*, recombinase polymerase amplification, CRISPR/Cas12a, one-tube assay, visual detection

## Abstract

*Bacilus cereus* and *Pseudomonas fluorescens* are major foodborne psychrotrophic bacteria posing global health and economic risks. *B. cereus* has a 23.8% food prevalence worldwide. *P. fluorescens* is a leading cause of spoilage in refrigerated products. Their rapid detection is crucial for food safety. However, existing detection methods often rely on open-tube operations, risking aerosol contamination. In this study, we developed two independent one-tube RPA-CRISPR/Cas12a visual detection assays for *B. cereus* and *P. fluorescens*. Using a physical separation design, the recombinase polymerase amplification (RPA) and CRISPR/Cas12a detection were pre-assembled in a single reaction tube. After incubation, a brief centrifugation combined the components for enclosed detection. This step is compatible with portable mini-centrifuges. The assays can be completed within 40 min at 37 °C, with results visualized directly under blue light. Both assays demonstrated good specificity against six common non-target pathogens. The visual detection limits were 5.1 × 10^1^ copies/μL for *B. cereus* and 2.1 × 10^1^ copies/μL for *P. fluorescens.* Each assay was applied to 14 types of real-world food samples (naturally contaminated and uncontaminated, confirmed by PCR), achieving 100% concordance with conventional PCR. The one-tube assays are tailored for psychrotrophic bacteria in refrigerated foods. They minimize aerosol contamination risk and provide a reliable solution for on-site cold-chain food safety monitoring.

## 1. Introduction

Psychrotrophic bacteria are defined as microorganisms capable of growing at refrigeration temperatures (≤7 °C), regardless of their optimum growth temperature [1]. When such microbes contaminate food and pose a risk of causing foodborne illnesses, they are classified as foodborne psychrotrophic bacteria, representing a persistent threat to the safety of refrigerated and cold-chain foods. *Pseudomonas fluorescens* and *Bacillus cereus* are two prominent representatives of significant concern in food safety. *P. fluorescens* is a dominant spoilage organism in refrigerated dairy and meat products, causing significant economic losses worldwide due to thermostable enzymes, biofilm formation, and quality defects such as slime, pigment production, and off-odors [2]. It is also an opportunistic pathogen capable of causing serious infections such as sepsis [3]. Globally, *B. cereus* exhibits a food prevalence of 23.8% based on a meta-analysis covering 98 studies [4], with particularly high contamination rates in Southern China reaching 55.8% in cereal flour and 45.7% in wheat/rice noodles [5]. It produces both emetic and diarrheal enterotoxins [6] and forms heat-resistant endospores, making it a leading cause of bacterial food poisoning [7]. Therefore, the development of detection technologies capable of early, rapid, and accurate identification of such foodborne psychrotrophic bacteria is crucial for the proactive prevention of food safety incidents and the protection of public health.

Current rapid detection of foodborne psychrotrophic bacteria primarily relies on molecular biology methods, such as polymerase chain reaction (PCR) and its derivative techniques. Although these methods significantly reduce detection time, their dependence on precise thermal cycling equipment and the need for skilled personnel limit their application in resource-limited or field settings [8]. To meet the demand for rapid, on-site testing without thermal cyclers, isothermal nucleic acid amplification technologies have been developed. These techniques enable rapid and efficient amplification of target nucleic acids at a constant temperature. Common isothermal amplification methods include recombinase polymerase amplification (RPA), rolling circle amplification (RCA), recombinase-mediated amplification (RAA), and loop-mediated isothermal amplification (LAMP) [9]. Among these, RPA is particularly notable for enabling exponential amplification of target sequences within 30 min at 37–42 °C [10]. The simplicity and low-temperature operation of RPA, while advantageous, can lead to compromised specificity through mechanisms such as non-specific amplification or contamination, impacting overall result reliability [11].

In recent years, Clustered Regularly Interspaced Short Palindromic Repeats (CRISPR) and associated protein systems have attracted significant interest in molecular diagnostics due to their operational simplicity, speed, high sensitivity, and strong specificity [12]. The Cas12a protein, in particular, has emerged as a powerful tool for specific nucleic acid detection owing to its efficient trans-cleavage activity upon target recognition [13]. However, when used alone, the Cas12a system exhibits limited sensitivity for detecting low-copy-number nucleic acid targets. Consequently, many studies have coupled it with isothermal amplification techniques: isothermal amplification is first employed for efficient pre-amplification to enhance sensitivity [14], after which the CRISPR/Cas12a system specifically recognizes the amplicons to ensure detection accuracy [15]. To date, the RPA-CRISPR/Cas12a method has emerged as a successful strategy for detecting a range of bacteria, such as *Haemophilus parasuis* [16], *Salmonella* [17], and *Staphylococcus aureus* [18]. However, conventional coupling strategies typically involve a “two-step” approach, where the RPA amplicon must be manually transferred to a separate CRISPR detection reaction. This open-tube step introduces a high risk of aerosol contamination and increases operational complexity, hindering its practical adoption for standardized on-site testing.

To overcome these limitations, we developed two independent one-tube RPA-CRISPR/Cas12a visual detection assays for *B. cereus* and *P. fluorescens*. Using a physical separation design, the assays integrate amplification and detection in a closed system, eliminating aerosol contamination risk associated with open-tube operations. To our knowledge, this is the first one-tube RPA-CRISPR/Cas12a assay specifically developed for *P. fluorescens*. For *B. cereus*, although one-tube assays have been reported [19], we identified and targeted a highly conserved region of the *nheB* gene distinct from previous studies, designing specific primers and crRNA optimized for our one-tube format. Both assays were validated using real-world cold-chain food samples, demonstrating their reliability in complex matrices. By providing a contamination-controlled solution tailored for psychrotrophic bacteria in refrigerated foods, this work addresses a critical gap in rapid on-site monitoring of cold-chain food safety.

## 2. Materials and Methods

### 2.1. Reagents and Materials

Tryptone and yeast extract were purchased from Thermo Fisher Scientific Oxoid (Waltham, MA, USA). The TIANamp Bacteria DNA Kit DP302 was obtained from Tiangen Biotech (Beijing, China), and the TwistAmp^®^ Basic RPA Kit (Catalog No. TABAS03KIT) was from BioMorey (Shanghai, China). The RNase inhibitor and LbCpf1 (Cas12a) nuclease were sourced from BBI (Shanghai, China) and Tolo Biotech (Shanghai, China), respectively. All primers, ssDNA reporters, and crRNAs were synthesized by Sangon (Shanghai, China). Detailed sequence information is provided in Table S1.

### 2.2. Bacterial Culture and DNA Extraction

Standard strains of *B. cereus* and *P. fluorescens* were cultured separately in LB liquid medium at 30 °C with shaking at 200 rpm for 12 h. Genomic DNA was extracted from a 1 mL aliquot of each overnight culture using the Tiangen Bacteria Genomic DNA Extraction Kit. DNA concentration was quantified using a Nanodrop 2000 (Thermo Fisher Scientific, Waltham, MA, USA), and samples were then stored at −20 °C until needed for further experiments. Prior to counting, bacterial cells were standardized by diluting overnight culture in fresh LB medium to OD600 = 1.0. The corresponding bacterial concentration was immediately determined by plate colony counting. Based on this value, a series of tenfold serial dilutions were prepared to obtain bacterial suspensions spanning concentrations from 10^6^ down to 10^0^ CFU/mL. Following extraction from 1 mL of each diluted suspension with the DNA Extraction kit, all genomic DNA samples were stored at −20 °C. In addition, genomic DNA samples from five other common foodborne pathogenic bacteria were used for specificity validation in this study. Relevant strain information and their culture conditions are listed in Table S2.

### 2.3. Design of Primers, ssDNA Reporters, and crRNAs

Specific genes for *B. cereus* and *P. fluorescens* were initially retrieved from the NCBI GenBank database. Based on criteria of intra-species conserved and inter-species specific, the *rpoD* gene (for *P. fluorescens*) and the *nheB* gene (for *B. cereus*) were ultimately selected as detection targets. Subsequently, the whole-genome sequences of 30 strains per target species were obtained from the database. Highly conserved regions within the selected genes were identified by nucleotide BLAST analysis against the NCBI GenBank database. Specific RPA primers targeting these conserved regions were then designed using Primer Premier 5.0 software, adhering to the following key parameters: a length of 30–35 nt, a GC content of 40–60%, a Tm of 50–70 °C, and an amplicon size under 500 bp, while also avoiding significant secondary structure or primer-dimer formation. The specificity of these primers was further verified using Primer-BLAST. Within the RPA amplicon, crRNAs for the CRISPR/Cas12a system were designed. All oligonucleotides used in this study, including RPA primers, crRNAs, and the single-stranded DNA (ssDNA) fluorescent reporters, were synthesized and HPLC-purified by Sangon (Shanghai, China). Detailed information is provided in Table S1.

### 2.4. RPA Reaction and CRISPR/Cas12a Detection

The RPA reaction mixture was prepared and assembled into the manufacturer-provided lyophilized pellet tube according to its instructions, containing 29.5 µL of 2 × Rehydration Buffer, 2.4 µL each of 10 µM forward and reverse primers, 2 µL of DNA template, 2.5 µL of magnesium acetate, and nuclease-free water to a final volume of 50 µL. To ensure thorough mixing, the sample underwent three sequential rounds of vortexing for 5 s and centrifugation for 5 s. The reaction system was then incubated at 37 °C in a metal bath for 20 min. Amplification products were visualized using a gel imaging system after being analyzed on a 2% agarose gel. The CRISPR/Cas12a detection reaction (20 µL) contained 2 µL of 10 × TOLO Buffer 3 (Tolo Biotech, Shanghai, China), 1 µL each of the 10 µM ssDNA reporter probe and 10 µM crRNA, 1 µL of 1 µM Cas12a nuclease, 1 U of RNase inhibitor, 2 µL of the RPA product, and DEPC-treated water (Sangon Biotech, Shanghai, China) to 20 µL. After vortex mixing and brief centrifugation, the reactions were transferred to a real-time PCR instrument for incubation and real-time fluorescence monitoring. Endpoint fluorescence could also be visualized with a blue-light transilluminator or quantified using a microplate reader (Tecan, Männedorf, Switzerland).

### 2.5. Establishment of the One-Tube RPA-CRISPR/Cas12a Detection Assay

To enable rapid detection and avoid aerosol contamination, one-tube RPA-CRISPR/Cas12a assays were developed. The procedure was as follows: A 15 µL mixture containing 14 µL of RPA premix and 1 µL of DNA template was placed at the bottom of a 0.1 mL tube (using optical flat-cap PCR tube, which provided sufficient adhesion to prevent premature droplet falling). Separately, a 5 µL CRISPR/Cas12a detection premix (containing 1 µL of 1 µM Cas12a nuclease, 1 µL of 10 µM crRNA, 1 µL of 10 µM ssDNA reporter probe, and 2 µL of 10× TOLO Buffer 3) was aliquoted onto the center of the tube cap. The tube was sealed and incubated at 37 °C in a metal bath for 20 min to allow RPA amplification with the tube placed horizontally and without shaking to prevent early mixing. It was then briefly centrifuged (using a manual pulse mini-centrifuge, which provided centrifugation for approximately 5 s) to combine the two compartments. The mixture was immediately transferred to a real-time PCR instrument. Fluorescence was monitored in real time at 37 °C using the FAM channel (excitation: 485 nm, emission: 520 nm) with data acquisition every minute. For endpoint detection, results could be qualitatively assessed by visualizing the reaction tube under a blue-light transilluminator, and images were captured using a smartphone. For quantification, the reaction mixture was transferred to a 96-well plate, and the endpoint fluorescence intensity was measured using a multifunctional microplate reader.

### 2.6. Evaluation of the Specificity and Sensitivity of One-Tube RPA-CRISPR/Cas12a Detection Assay

To validate the specificity and sensitivity of the two RPA-CRISPR/Cas12a one-tube detection assays, we conducted the following experiments. First, genomic DNA from *Bacillus cereus*, *Pseudomonas fluorescens*, *Staphylococcus aureus*, *Listeria monocytogenes*, *Salmonella typhimurium*, *Escherichia coli O157:H7*, and *Vibrio parahaemolyticus* were used as templates, with double-distilled water as a blank control, to assess the detection specificity of both assays for the target bacteria (*P. fluorescens* or *B. cereus*). Detection results were assessed qualitatively by visualizing fluorescence under a blue-light transilluminator and quantitatively by measuring fluorescence intensity with a microplate reader.

Next, sensitivity testing was conducted. Genomic DNA from *P. fluorescens* and *B. cereus* was extracted, and serially diluted tenfold with double-distilled water to generate standard templates at concentrations ranging from 5.1 × 10^6^ to 5.1 × 10^0^ copies/μL for *B. cereus* and from 2.1 × 10^6^ to 2.1 × 10^0^ copies/μL for *P. fluorescens*. Each dilution was tested using the corresponding one-tube assay, with double-distilled water included as the blank control. After the reaction, fluorescence was observed directly under a blue-light transilluminator and quantified using a microplate reader to determine the limit of detection (LOD) for each assay.

### 2.7. Application of the One-Tube RPA-CRISPR/Cas12a Detection Assay in Spiked Samples

To evaluate the practical applicability and sensitivity of the two one-tube RPA-CRISPR/Cas12a assays, artificial spiking experiments were performed using commercially purchased pork and chicken as food matrices. The two assays were developed independently, one specifically targeting *B. cereus* and the other targeting *P. fluorescens*. Prior to spiking, conventional PCR was conducted to confirm the absence of *B. cereus* in the pork samples and of *P. fluorescens* in the chicken samples, using gene-specific primers (Table S1) and standard cycling conditions (Table S3). Subsequently, pure cultures of *B. cereus* and *P. fluorescens* were serially diluted to obtain bacterial suspensions with concentrations ranging from 3.2 × 10^6^ to 3.2 × 10^0^ CFU/mL and from 2.6 × 10^6^ to 2.6 × 10^0^ CFU/mL, respectively. The spiked samples were prepared as follows: A 1 g portion of each food sample (pork or chicken) was homogenized with 10 mL of LB liquid medium to create a homogenate. Then, 100 µL of this homogenate was thoroughly mixed with 1 mL of the corresponding bacterial suspension (pork homogenate with *B. cereus* suspension; chicken homogenate with *P. fluorescens* suspension) to yield the final spiked sample. Nucleic acids were extracted from all spiked samples and controls using the TIANamp Genomic DNA Extraction Kit. The extracted DNA was then used as the template in the corresponding one-tube RPA-CRISPR/Cas12a reaction, with 1 µL of DNA added per reaction. Nuclease-free water was included as a no-template control (NTC). Results were evaluated qualitatively by visualization under a blue-light transilluminator and quantitatively by measuring the endpoint fluorescence intensity using a microplate reader (λex/λem = 530/560 nm).

### 2.8. Application of the One-Tube RPA-CRISPR/Cas12a Detection Assay in Real Samples

To evaluate the practical performance of the two developed one-tube RPA-CRISPR/Cas12a detection assays, a variety of food samples were collected from a local market, including vegetables, chicken, pork, beef, milk, cheese, salad, pureed fruit, salmon, sausage, braised meat, rice, pasta, ice cream, shellfish, ham, shrimp, lettuce, fruit slices, fruit juice, eggs, and sandwiches. For each sample, 25 g (or 25 mL for liquids) was mixed with 225 mL of LB medium and incubated at 30 °C with shaking for 12 h for enrichment. Total bacterial genomic DNA was extracted using a boiling method. Briefly, a 1 mL aliquot of enriched culture was centrifuged (12,000× *g*, 2 min), and the pellet was resuspended in 100 µL of nuclease-free water. The suspension was heated at 95 °C for 10 min, cooled on ice (2 min), and centrifuged again (12,000× *g*, 5 min). Finally, supernatant containing the DNA was collected. For the Gram-positive *B. cereus*, the cell pellet was treated with lysozyme (TIANGEN Biotech, Beijing, China) (20 mg/mL) at 37 °C for 10 min prior to the heating step. The extracted DNA was then tested with the two one-tube RPA-CRISPR/Cas12a assays, one targeting *B. cereus* and the other targeting *P. fluorescens*. To validate the accuracy of the assays, all samples were tested in parallel using conventional PCR assays specific for the corresponding target genes. The detection results obtained from the one-tube assays were compared with those from PCR to determine their reliability.

### 2.9. Statistical Analysis

Fluorescence intensity was measured using a multifunctional microplate reader (Tecan, Männedorf, Switzerland) with excitation/emission wavelengths set at 530/560 nm. Data were obtained from three independent biological replicates, each with three technical replicates, and are presented as mean ± SEM. Statistical analyses and graphing were performed using Origin 2022 (OriginLab, Northampton, MA, USA). For multiple comparisons against the negative control, one-way ANOVA followed by Dunnett’s post hoc test was applied, with *p*-values adjusted for multiple comparisons. The limit of detection (LOD) was defined as the lowest concentration that produced a fluorescence signal significantly higher than the negative control (Dunnett’s test, adjusted *p* < 0.05), with all replicates at that concentration meeting this criterion and no lower concentration showing a significant difference from the control. Significance levels are indicated as follows: * *p* ≤ 0.05, ** *p* ≤ 0.01, *** *p* ≤ 0.001.

## 3. Results

### 3.1. One-Tube RPA-CRISPR/Cas12a Assay Detection Principle

**Figure 1:**
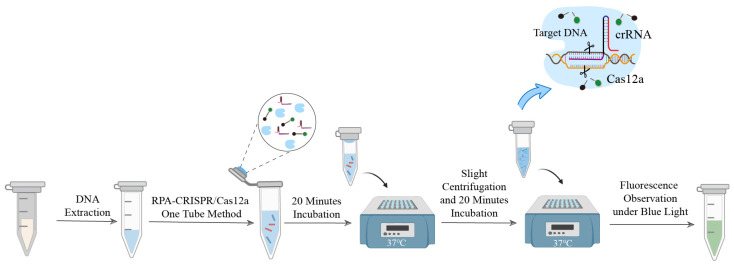
Schematic of two one-tube RPA-CRISPR/Cas12a detection assays.

The working principle of the one-tube RPA-CRISPR/Cas12a detection assay is illustrated in Figure 1. The assay features a physically separated, integrated design comprising two components: the RPA amplification system at the bottom of the tube and the CRISPR/Cas12a detection system in the cap. Briefly, the CRISPR/Cas12a detection mix is pre-dispensed onto the inner surface of the tube cap, while the sample DNA is added to the pre-prepared RPA reaction mix at the tube bottom. Following incubation at 37 °C for 20 min to allow isothermal target amplification, the tube is briefly centrifuged and shaken. This combines the two compartments, initiating the CRISPR/Cas12a detection reaction. Detection relies on the trans-cleavage activity of Cas12a. If target DNA is present, the RPA amplicons bind to the crRNA-guided Cas12a complex, activating its nonspecific cleavage activity. This activity cleaves the free ssDNA reporter probe (5′-HEX-(TTTT)_3_-BHQ1-3′), separating the HEX fluorophore from the BHQ1 quencher and generating a fluorescent signal. In the absence of the target, Cas12a remains inactive, and no fluorescence is produced. This one-tube design integrates amplification and detection, eliminating manual amplicon transfer and the risk of aerosol contamination. It thereby simplifies the workflow and is well-suited for rapid on-site detection.

### 3.2. Feasibility Analysis and Condition Optimization of the One-Tube RPA-CRISPR/Cas12a Detection Assay

**Figure 2:**
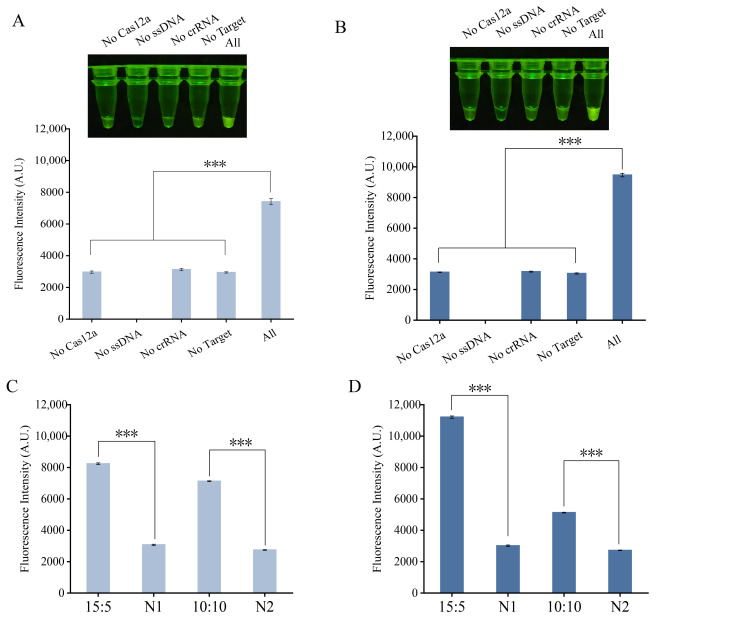
Feasibility verification and reaction optimization of the one-tube RPA-CRISPR/Cas12a assay. (**A**) Feasibility analysis of the *Bacillus cereus* detection system. Visual results under blue light (top) and corresponding fluorescence intensities (bottom). (**B**) Feasibility analysis of the *Pseudomonas fluorescens* detection system. Visual results and corresponding fluorescence intensities. (**C**) Optimization of RPA-to-CRISPR volume ratio for *B. cereus* detection. Comparison of fluorescence intensity between two volume ratios: 10 μL: 10 μL and 15 μL: 5 μL. (**D**) Optimization of RPA-to-CRISPR volume ratio for *P. fluorescens* detection. Fluorescence intensity comparison between the two volume ratios. N, negative control (no template added). *** *p* ≤ 0.001.

To validate the feasibility of the one-tube RPA-CRISPR/Cas12a assays, a control experiment consisting of five reaction systems was designed: one complete reaction mixture and four depletion controls, each lacking one key component (Cas12a nuclease, crRNA, ssDNA reporter probe, or RPA amplicon). As shown in Figure 2A (for *B. cereus*) and Figure 2B (for *P. fluorescens*), a strong fluorescent signal was observed only in the complete reaction, while the omission of any single key component resulted in a marked reduction or complete loss of fluorescence. One-way ANOVA followed by Dunnett’s post hoc test confirmed that each depletion control exhibited significantly lower fluorescence than the complete reaction. These results confirm that Cas12a, crRNA, the ssDNA reporter, and RPA amplicons are all indispensable for signal generation, thereby verifying the basic feasibility of the integrated assay.

To optimize detection performance, we systematically evaluated the volume ratio between the RPA amplification and CRISPR/Cas12a detection systems. The ratio was chosen based on two key considerations: (1) ensuring sufficient RPA volume for adequate target amplification while minimizing non-specific background, and (2) keeping the CRISPR detection droplet small enough to remain stable on the tube cap and minimize handling loss. Two representative ratios were compared: RPA:CRISPR at 10 µL: 10 µL (balanced design) and 15 µL: 5 µL (RPA-amplification-favored design). This comparison reflects the difference between balanced allocation and amplification-priority strategies, an approach commonly employed in one-tube CRISPR assays [20,21]. Fluorescence measurements showed that the 15 µL: 5 µL ratio produced the strongest signal and highest signal-to-noise ratio for both target bacteria (Figure 2C,D). This is likely attributable to the higher yield of RPA amplicons from the 15 µL amplification. When mixed with the smaller (5 µL) CRISPR detection volume, the local concentration of target amplicons in the final reaction mixture is substantially increased. This higher local concentration promotes more efficient target recognition by the Cas12a-crRNA complex and accelerates the trans-cleavage of the reporter probe, culminating in the strongest fluorescence signal within the given reaction time.

### 3.3. Specificity and Sensitivity of the One-Tube RPA-CRISPR/Cas12a Detection Assay

**Figure 3:**
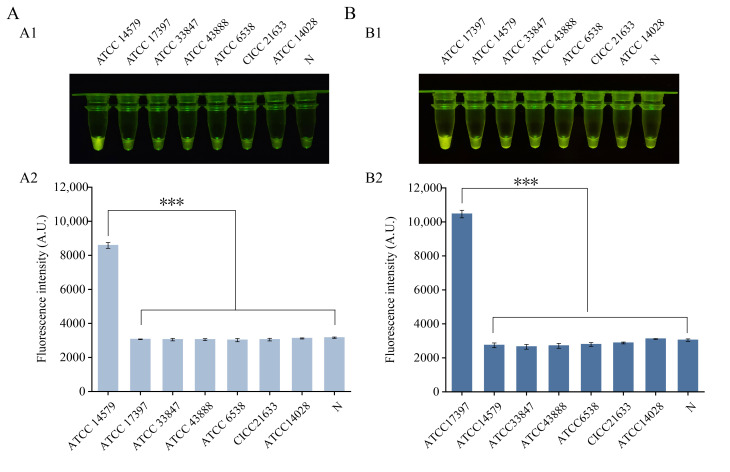
Specificity of the one-tube RPA-CRISPR/Cas12a detection assays. (**A**) Fluorescence visualization results and fluorescence intensity values for *Bacillus cereus*. Visual fluorescence readout (**A1**) and the corresponding fluorescence intensity (**A2**). (**B**) Fluorescence visualization results and fluorescence intensity values for *Pseudomonas fluorescens*. Visual fluorescence readout (**B1**) and the corresponding fluorescence intensity (**B2**). N, negative control (no template added). *** *p* ≤ 0.001.

The specificity of each assay was validated using genomic DNA from seven foodborne pathogens, which included the target bacterium (*B. cereus* or *P. fluorescens*) and six non-target species. A clear increase in fluorescence, visible to the naked eye, was observed only in reactions containing the target DNA. In contrast, no significant fluorescence signal was detected for any of the six non-target pathogens tested or the double-distilled water negative control (Figure 3A,B). These results demonstrate that both assays can distinguish their respective target bacteria from the panel of common foodborne pathogens evaluated in this study. This specificity is attributed to the bioinformatic selection of target genes (*nheB* for *B. cereus* and *rpoD* for *P. fluorescens*), which are highly conserved within the target species yet exhibit sequence divergence from non-target species. The design of specific RPA primers and crRNAs ensures precise recognition and amplification of the target region, minimizing off-target binding.

**Figure 4:**
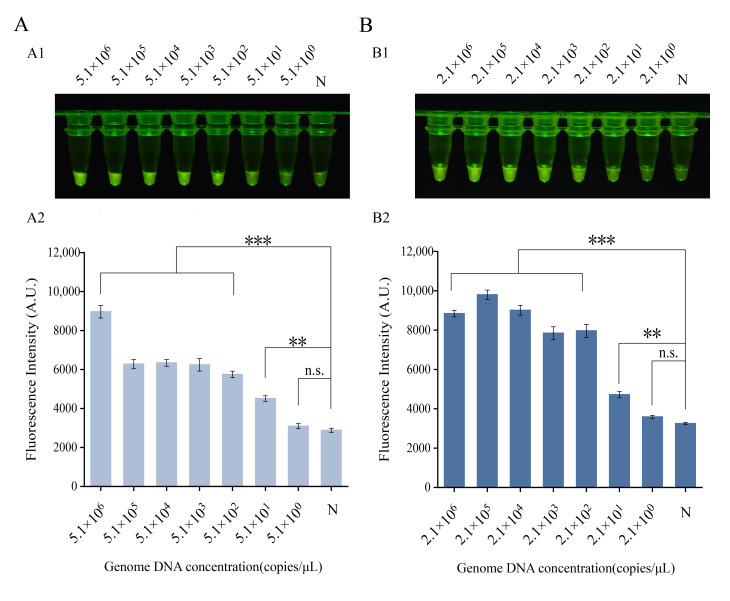
Sensitivity of the one-tube RPA-CRISPR/Cas12a detection assays. (**A**) Fluorescence visualization results and fluorescence intensity values for *Bacillus cereus*. Visual fluorescence readout (**A1**) and the corresponding fluorescence intensity (**A2**). (**B**) Fluorescence visualization results and fluorescence intensity values for *Pseudomonas fluorescens*. Visual fluorescence readout (**B1**) and the corresponding fluorescence intensity (**B2**). N, negative control (no template added). ** *p* ≤ 0.01, *** *p* ≤ 0.001.

Analytical sensitivity was assessed using serially diluted target genomic DNA. For the *B. cereus* assay, fluorescence signals were significantly above the negative control across a concentration range from 5.1 × 10^6^ down to 5.1 × 10^1^ copies/μL. At 5.1 × 10^0^ copies/μL, the signal was indistinguishable from the background, establishing a visual limit of detection (LOD) of 5.1 × 10^1^ copies/μL (Figure 4A). Similarly, for the *P. fluorescens* assay, the visual LOD was determined to be 2.1 × 10^1^ copies/μL, as no visible fluorescence was observed below this concentration (Figure 4B). In summary, both one-tube RPA-CRISPR/Cas12a assays developed here achieve highly specific and sensitive detection of their respective target bacteria at low concentrations.

### 3.4. One-Tube RPA-CRISPR/Cas12a Assay Detection of Spiked Samples

**Figure 5:**
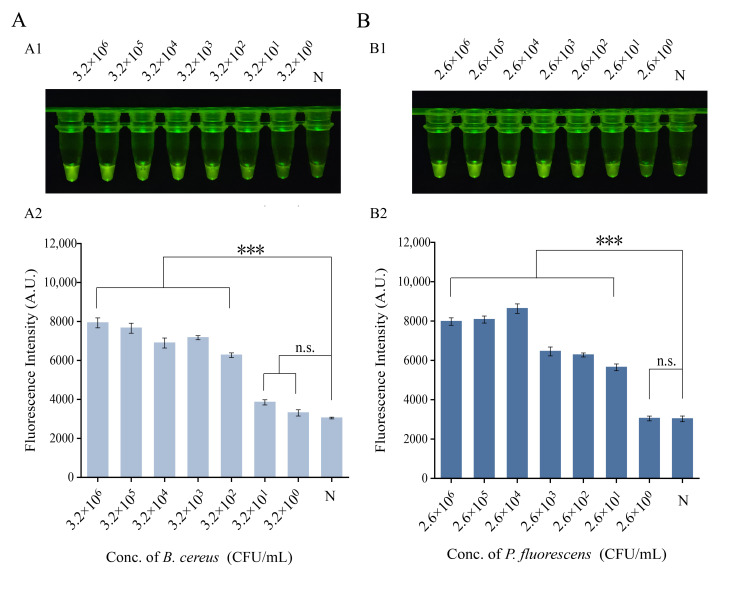
Applicability of the one-tube RPA-CRISPR/Cas12a assay for detecting artificially spiked samples. (**A**) The detection results of pork samples contaminated with *Bacillus cereus* at different concentrations. Visual fluorescence readout (**A1**) and the corresponding fluorescence intensity (**A2**). (**B**) The detection results of chicken samples contaminated with *Pseudomonas fluorescens* at different concentrations. Visual fluorescence readout (**B1**) and the corresponding fluorescence intensity (**B2**). N, negative control (no template added). *** *p* ≤ 0.001.

To evaluate the interference resistance and detection performance of the two established one-tube RPA-CRISPR/Cas12a assays in complex food matrices, artificial spike experiments were conducted using representative foods. Pork and chicken were selected as the test matrices due to their common association with *B. cereus* and *P. fluorescens* contamination, respectively. Prior to spiking, the conventional PCR method confirmed the absence of the target bacteria in these food samples. The food homogenates were artificially contaminated with serially diluted target bacterial suspensions. Genomic DNA was extracted from the spiked samples and analyzed using the respective assays. For pork spiked with *B. cereus*, fluorescence intensity was significantly higher than the blank control (*p* < 0.05) at concentrations ≥ 3.2 × 10^2^ CFU/mL, with a distinct green fluorescence visible under blue light (Figure 5A). Similarly, for chicken spiked with *P. fluorescens*, the consistent detection threshold was 2.6 × 10^2^ CFU/mL, at which the signal showed a statistically significant difference from the negative control (Figure 5B). These results demonstrate that both one-tube assays retain their detection efficacy in the presence of complex food matrices, highlighting their robustness and practical applicability for food safety monitoring.

### 3.5. One-Tube RPA-CRISPR/Cas12a Assay Detection of Real Samples

**Figure 6:**
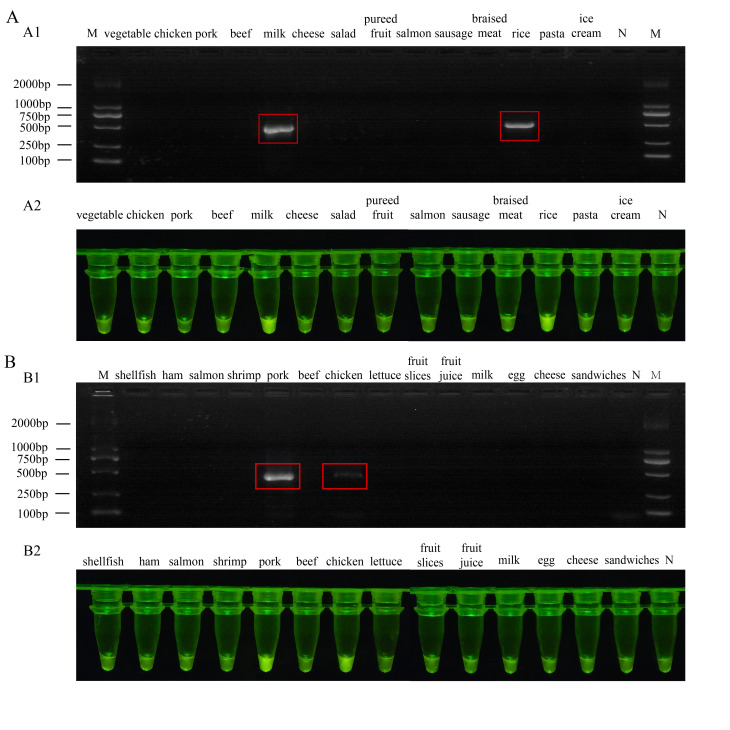
Comparison of the detection results of the one-tube RPA-CRISPR/Cas12a assay with PCR method. (**A**) PCR results of *Bacillus cereus* in various refrigerated food samples (**A1**). Corresponding fluorescence detection results (**A2**) of *B. cereus*. (**B**) PCR results of *Pseudomonas fluorescens* in various refrigerated food samples (**B1**). Corresponding fluorescence detection results (**B2**) of *P. fluorescens*. The red square indicates the sample that tested positive for the target. M, DL2000 DNA marker; N, negative control.

The reliability of the one-tube RPA-CRISPR/Cas12a assays was further assessed by testing them on actual food samples. We selected 14 food types prone to *B. cereus* contamination and another 14 prone to *P. fluorescens* contamination, testing each set with its corresponding pathogen-specific assay. Results from the *B. cereus* assay showed positive signals for refrigerated rice and milk samples, whereas the other 12 foods and the blank control exhibited no detectable fluorescence (Figure 6A). Similarly, the *P. fluorescens* assay yielded positive results for chicken and pork samples, with all remaining samples testing negative (Figure 6B). Importantly, no false-positive signals were observed for any negative sample. All findings were confirmed by conventional PCR, and the results showed 100% concordance for each assay (14/14 samples for *B. cereus* and 14/14 samples for *P. fluorescens*) between the one-tube assays and PCR. In summary, the assays demonstrated high accuracy and reliability in detecting two distinct foodborne psychrotrophic bacteria across a variety of complex food matrices. Their ability to correctly identify contaminated samples without cross-reactivity highlights their strong potential for practical food safety monitoring.

## 4. Discussion

*P. fluorescens* and *B. cereus* are major foodborne psychrotrophic pathogens posing significant risks to refrigerated foods. Current detection methods for these pathogens have notable limitations: culture methods are time-consuming (2–3 days), PCR requires thermal cyclers, and existing RPA-CRISPR assays often rely on open-tube two-step operations that risk aerosol contamination and false positives [19,22]. To address these challenges, we developed two independent one-tube RPA-CRISPR/Cas12a visual assays for *P. fluorescens* and *B. cereus*, including the first one-tube assay for *P. fluorescens* and a parallel system for both psychrotrophic pathogens. Both assays achieved visual detection limits of 5.1 × 10^1^ copies/μL for *B. cereus* and 2.1 × 10^1^ copies/μL for *P. fluorescens* within 40 min at 37 °C. For field deployment, the assay requires only minimal portable equipment: a battery-operated metal bath, a handheld blue light lamp, and a manual mini-centrifuge (or brief hand-flicking). Fluorescence results are visualized directly by eye. Compared to other rapid detection technologies, our assays offer a favorable combination of one-tube operation, visual readout, portability, and sensitivity (Table 1).

During method development, occasional false positives in preliminary two-step assays due to aerosolized amplicons motivated our one-tube design. In the final one-tube format, all negative controls showed no detectable fluorescence across multiple independent runs, with stable background fluorescence values (mean ± SD) of 2882 ± 144 RFU for *B*. *cereus* assays and 3247 ± 80 RFU for *P*. *fluorescens* assays (coefficient of variation < 15%). To further minimize cross-contamination risk, strict laboratory practices were implemented, including physical separation of pre- and post-amplification areas, dedicated pipettes for each zone, and routine use of nucleic acid decontamination solutions. Using this assay, we assessed its practical utility with real food samples selected based on known contamination risks: 14 food types commonly associated with *B. cereus* (e.g., rice, milk, pasta) and 14 types prone to *P. fluorescens* contamination (e.g., chicken, pork, fish, vegetables) collected from local markets. Each sample was tested in parallel with PCR, and the assays demonstrated 100% concordance with PCR results, confirming their reliability in complex food matrices. Nevertheless, the sample size per matrix was limited and may not fully represent all refrigerated food categories. Larger-scale studies with broader sampling are therefore required to establish generalizability.

Regarding specificity, the assays showed good exclusivity against six common non-target pathogens. However, the *B. cereus* assay targeting *nheB* cannot discriminate members of the *B. cereus sensu lato* group, such as *Bacillus thuringiensis*, due to high genetic similarity. Similarly, the *P. fluorescens* assay targeting *rpoD* may theoretically amplify closely related species, including *Pseudomonas antarctica* and *Pseudomonas rhodesiae*, within the *P. fluorescens* complex. From a food safety perspective, this theoretical cross-reactivity represents a conservative screening advantage rather than a methodological flaw, as *nheB*-positive *B. thuringiensis* strains are enterotoxigenic and related *Pseudomonas* species share similar ecological niches and spoilage potential.

Several limitations should be acknowledged. Although three independent biological replicates (each with three technical replicates) satisfy technical validation requirements, the limited sample size restricts statistical robustness, and larger studies are needed to establish confidence intervals. The current single-target format limits throughput, and the assay detects DNA from both viable and non-viable cells, potentially overestimating risk in processed foods. The assay presently relies on liquid reagents requiring cold-chain transport. Moreover, the reported 40 min detection time refers strictly to the post-enrichment assay; at extremely low contamination levels, optional short-term enrichment (2–5 h) or rapid filtration (5–10 min) may be applied.

Future work will focus on: (i) integrating PMAxx pretreatment for viable-cell discrimination [23]; (ii) developing ambient-stable, ready-to-use lyophilized reagent formats [24]; (iii) enabling multiplex detection via crRNA arrays [25] or microfluidic integration [26]; (iv) validating performance using larger and more diverse sample cohorts; and (v) conducting systematic cross-validation with closely related species within the *B. cereus sensu lato* group and *P. fluorescens* complex; and (vi) integrating smartphone-based image analysis or portable fluorescence readers for objective, quantitative readouts. Owing to the modular primer and crRNA design [27], the assay can be readily adapted to other foodborne pathogens, supporting its evolution into a versatile, field-deployable tool for cold-chain food safety monitoring.

**Table 1:** Comparison of the proposed one-tube RPA-CRISPR/Cas12a assay with existing detection methods.

No.	Analytical Method	Target	Sensitivity	Time	Key Instrument Requirements	Reference
1	PCR	*Pseudomonas fluorescens*	10^5^ CFU/mL	2 h	PCR machine, Electrophoresis apparatus	[22]
2	droplet digital PCR	*Bacillus cereus*	log 1.01 cell/mL	80 min	Droplet generator, PCR thermal cycler, Droplet reader	[28]
3	PMA-LAMP	*Bacillus cereus*	1.16 × 10^2^ CFU/mL	80 min	Real-time PCR instrument	[23]
4	Fluorescent sensor	*Bacillus cereus*	5.9 × 10^2^ CFU/mL	3.5 h	PCR machine, Multifunctional microplate reader, Constant temperature device	[29]
5	Electrochemical apta-sensor	*Bacillus cereus*	1 CFU/mL	15 min	Portable PalmSens4 potentiostat/galvanostat/impedance analyzer	[30]
6	RPA-LF	*Bacillus cereus*	1.0 × 10^2^ CFU/mL	15 min	Constant temperature device	[31]
7	RPA-CRISPR/ Cas12a	*Bacillus cereus*	10^2^ CFU/mL 10^−2^ ng/μL (gDNA)	40 min	Constant temperature device, Blue light transilluminator	[19]
8	One-tube RPA-CRISPR/ Cas12a	*Pseudomonas fluorescens/* *Bacillus cereus*	2.1 × 10^1^ copies/µL/ 5.1 × 10^1^ copies/µL	40 min	Metal bath, Blue light lamp	This study

## 5. Conclusions

In this study, we established two independent one-tube RPA-CRISPR/Cas12a assays, including the first assay targeting the *nheB* gene of *B*. *cereus* and the first assay for *P*. *fluorescens*. These assays provide specific detection tools for two major psychrotrophic pathogens with distinct contamination profiles in refrigerated foods. By integrating physical separation with portable equipment (a metal bath and a blue light lamp), the assays enable on-site detection while minimizing aerosol contamination risk. The visual detection limits are 5.1 × 10^1^ copies/μL for *B. cereus* and 2.1 × 10^1^ copies/μL for *P. fluorescens*, achieved within 40 min. Successful validation with real food samples demonstrates the assays’ practical reliability. Additionally, the modular design of primers and crRNA makes the assay adaptable for detecting other foodborne pathogens. This work provides a strategic tool for monitoring cold-chain food safety, representing a shift from laboratory-based testing to field-deployable solutions.

## Data Availability

The original contributions presented in the study are included in the article/Supplementary Materials. Further inquiries can be directed to the corresponding author.

## Supplementary Materials

The following supporting information can be downloaded at https://www.mdpi.com/article/10.3390/foods15061059/s1: Table S1. Recombinase polymerase amplification primers, crRNAs, and reporter sequences used in this study; Table S2. Culture conditions and sources of bacterial strains; Table S3. PCR reaction system and procedure.
