# Supplementary material for: One-Tube RPA-CRISPR/Cas12a Assays for Rapid and Visual Detection of Pseudomonas fluorescens and Bacillus cereus
Source: Foods. 2026 Mar 17;15(6):1059. doi: 10.3390/foods15061059 (PMC13025563; doi:10.3390/foods15061059)
Supplement: Supplementary file 1 [file foods-15-01059-s001.zip › foods-4120863-supplementary.pdf]

Supplementary Material

Table S1. Recombinase polymerase amplification primers, crRNAs, and reporter sequences used in this study.

Name	Sequences (5'-3')	Amplicon length (bp)
B-RPA-F	GTAATTCAGCACCAAGATACAGCTAGAGGA	289
B-RPA-R	CCTTGAAGTTTTGCGTATCCGAAGTCATTT	
B-PCR-F	AATGCGAAGCAATGGTTAGA	466
B-PCR-R	CTACTCCTAGCGGTGTTCCT	
P-RPA-F	AACCGTGGCCTGCAATTCCTTGACCTGATC	326
P-RPA-R	GGCTCTTTAGCGTCTTCAATACCTTGCGGA	
P-PCR-F	CGAAGAAAGAGATGGTTGAA	443
P-PCR-R	GAAGTCACCCAGATGGGAGT	
B-crRNA	AAUUUCUACUGUUGUAGAUCAUCAACUGCA GCAACUAAAGUA	-
P-crRNA	AAUUUCUACUGUUGUAGAUCCGGCAGATGT TGCAGGAAATGC	-
Reporter	5'-HEX-TTTTTTTT-BHQ1-3'	-

Table S2. Culture conditions and sources of bacterial strains.

Bacterial species	Strains	Culture medium	Temperature (°C)	Time (h)	Source*
Bacillus cereus	ATCC14579	LB	30	12	a
Pseudomonas fluorescens	ATCC17397	LB	30	12	a
Vibrio parahaemolyticus	ATCC33847	BHI	37	12	b
Escherichia Coli O157:H7	ATCC43888	LB	37	12	c
Staphylococcus aureus	ATCC6538	TSB	37	12	d
Listeria monocytogenes	CICC21633	BHI	37	12	e
Salmonella typhimurium	ATCC14028	BHI	37	12	e

*a, Beijing biobw Biotechnology; b, Beijing Bena Culture Collection; c, Baosai Biotechnology; d, Guangdong Microbial Culture Collection Center; and e, China Centre of Industrial Culture Collection

Table S3. PCR reaction system and procedure.

PCR reaction system	20 μL	PCR reaction procedure			
2×Taq PCR Mix	10 μL		Temperature	Time	
		Predenaturation	95°C	3 min	
Forward primer	1 μL (10 μM)				
		Denaturation	95°C	30 s	33 cycles
		Annealing	55°C	30 s	
Reverse primer	1 μL (10 μM)				
		Extension	72°C	60 s	
ddH_2O	7 μL	Final extension	72°C	10 min	
DNA	1 μL				
